# Successful Bypass Surgery in a Healthy 24-Year-Old Male with Peripheral Artery Disease

**DOI:** 10.7759/cureus.3010

**Published:** 2018-07-20

**Authors:** Kishan J Padalia, Michael J Muehlberger

**Affiliations:** 1 College of Medicine, University of Central Florida College, Orlando, USA; 2 Vascular Specialists of Central Florida, Orlando Regional Medical Center, Orlando, USA

**Keywords:** peripheral artery disease, atherosclerosis, premature, young adult, bypass

## Abstract

Approximately 40% of peripheral artery disease cases occur in individuals less than 50 years old. Diagnosis is often delayed due to fewer risk factors, atypical presentation, and symptoms attributed to a benign cause. We present an unusual case of an otherwise healthy 24-year-old male presenting with unilateral, intermittent claudication (IC) due to diffuse atherosclerotic disease in his left femoral arteries. Lifestyle-limiting symptoms caused by a four-year delay in diagnosis were improved with successful left femoropopliteal bypass. We use this case to review the differential diagnosis for IC and recommend early revascularization in young patients with severe disease and few comorbidities.

## Introduction

There are approximately 202 million individuals living with peripheral artery disease (PAD), defined by an ankle-brachial index (ABI) of less than 0.9, of which an estimated 84 million (42%) are below the age of 50 and an estimated 14 million (7%) are between the ages of 25 and 29 [1]. Current guidelines for PAD management and treatment are informed predominantly by studies in patients 40 years and older, and no epidemiological studies have investigated the prevalence of PAD in individuals younger than 25 years of age. Here we present the case of an otherwise healthy 24-year-old PAD patient with a four-year history of lifestyle-limiting claudication who underwent successful bypass surgery.

## Case presentation

A 24-year-old male presented with severe throbbing pain extending from the distal half of his left thigh to his left foot. The pain initially began four years prior, was mild, and was triggered by walking one-half to one mile. The pain progressed and is now triggered by walking one block and relieved by several minutes of rest. He delayed medical evaluation, because he believed he was having muscle cramps. He denied any history of chest pain, palpitations, shortness of breath, lower extremity swelling, skin discoloration, trauma, or prenatal/birth complications. He had no personal or family history of hypertension, hyperlipidemia, diabetes mellitus, deep vein thrombosis, hypercoagulability, malignant neoplasms, or autoimmune disorders. The family history was also negative for PAD and myocardial infarction. He was taking no medications at the time of evaluation. He does not consume alcohol and has never smoked cigarettes or used illicit drugs.

**Figure 1:**
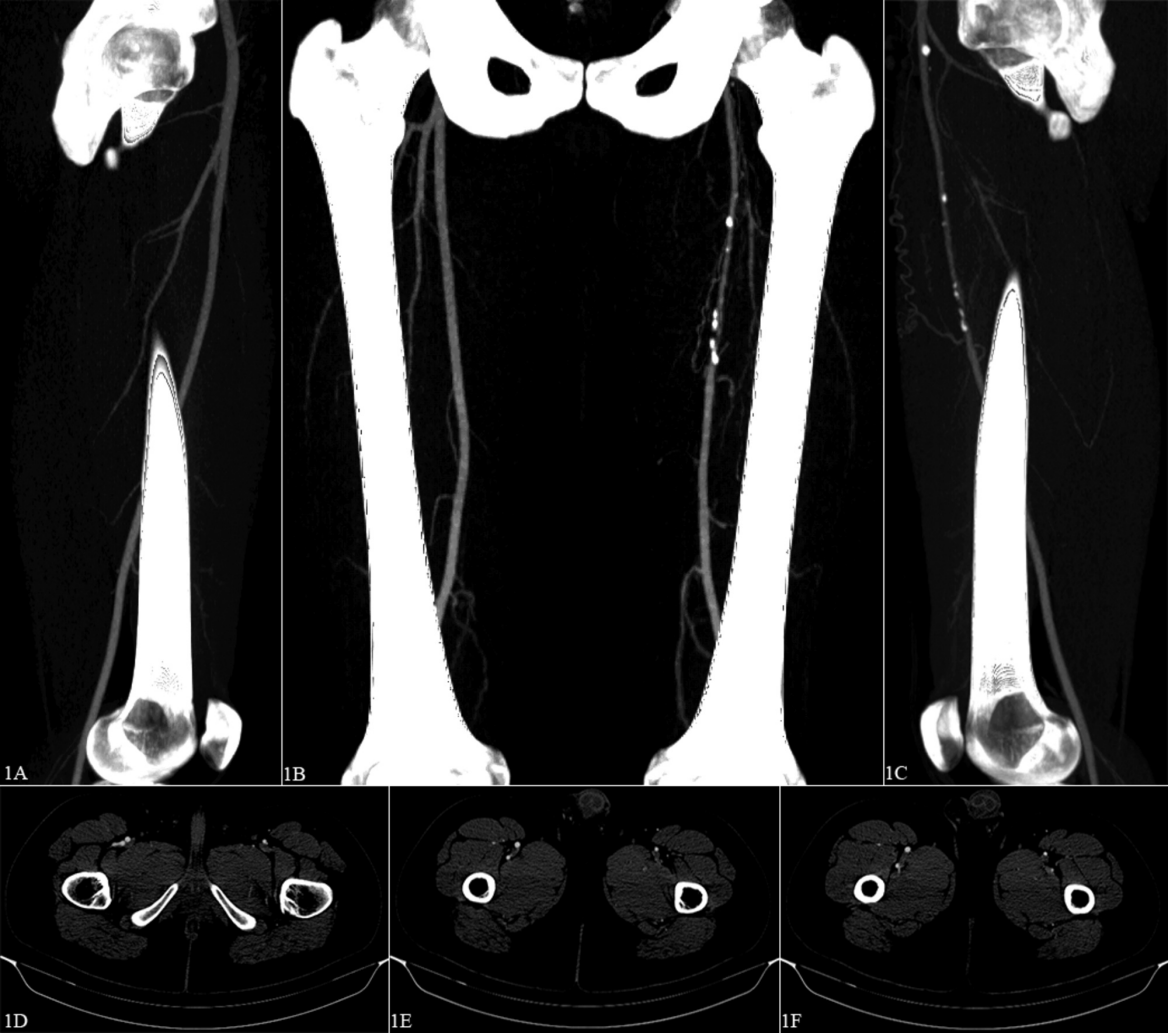
Contrast-enhanced computed tomography angiogram of abdomen and lower extremities. (A, C) Sagittal view of right and left above-knee lower extremity, respectively, and (B) coronal view of bilateral above-knee lower extremity. These views show a significantly diminutive left common femoral artery and partial occlusion of the left proximal to mid superficial femoral artery with diffuse calcifications. Collateralization with distal reconstitution is evident. (D-F) Axial views show complete occlusion of the left proximal profunda femoris artery (D, E) with distal reconstitution (F).

Physical examination revealed a heart rate of 72 beats per minute, left brachial blood pressure of 114/74 mmHg, and body mass index of 25.1 kg/m^2^. Lower extremities showed no pigment changes, edema, tenderness, and had full range of motion. The right femoral, popliteal, and posterior tibial pulses were palpable 2+. The left femoral artery was palpable 1+. The left popliteal, posterior tibial, and bilateral dorsalis pedis pulses were nonpalpable. The left popliteal and posterior tibial arteries had a weak, biphasic Doppler signal and the bilateral dorsalis pedis arteries had no appreciable Doppler signal. No carotid or abdominal bruits were noted, and the remainder of the physical exam was unremarkable. Laboratory values were within the normal range: total cholesterol 161 mg/dL, high-density lipoprotein 58 mg/dL, triglycerides 52 mg/dL, low-density lipoprotein 90 mg/dL, hemoglobin (Hb) 16.1 g/dL, platelet 218,000/uL, creatinine 0.79 mg/dL, glucose 80 mg/dL, HbA1c 5.2%, prothrombin time 12.8 seconds, and international normalized ratio 1.0. Left ABI was 0.76 at rest and 0.40 after five minutes of exercise. Arterial duplex demonstrated biphasic waveforms from the left common and profunda femoris arteries with no stenosis. The left superficial femoral artery had bi/monophasic waveforms with >75% stenosis at the proximal thigh. The popliteal and tibial arteries had bi/monophasic flow without stenosis. A computed tomography (CT) angiogram with contrast and bilateral lower extremity runoff demonstrated normal arteries of the abdomen and right lower extremity (Figure 1A-C). The left common femoral artery was significantly smaller than the right but otherwise normal. The left proximal to mid superficial femoral artery was severely diseased with extensive calcifications causing near-complete occlusion with distal reconstitution (Figure 1A-C). The left proximal profunda femoris artery was completely occluded with lack of flow for the first 45 mm with distal reconstitution (Figure 1D-F). The left popliteal, tibial, peroneal, and dorsalis pedis arteries were normal with excellent runoff. There were no signs of embryological abnormalities.

**Figure 2:**
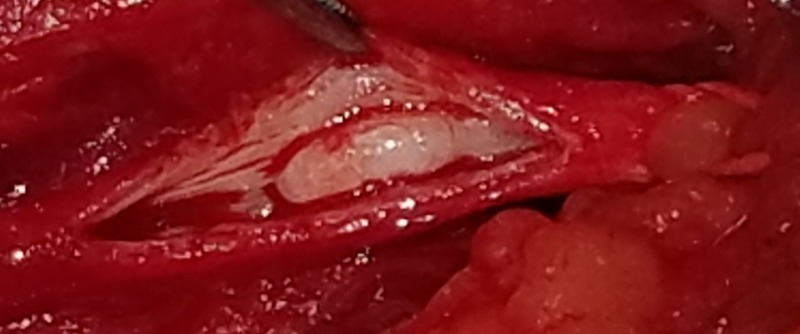
Gross image of a calcified lesion in the left proximal profunda femoris artery.

Surgical exploration confirmed that the left common femoral artery was diminutive but soft with a palpable pulse. The origin of the profunda femoris artery was obstructed with a smooth, hard lesion (Figure 2), which was removed with local endarterectomy and confirmed by pathology as a calcified plaque. The distal profunda femoris was followed and was found to be extensively full of the calcified plaque with no back bleeding, so a profundaplasty or complete profundal bypass was decided against, and a bovine pericardial patch was placed. Next, the left great saphenous vein was harvested and used for a left femoral to left above-knee popliteal artery bypass. A 2+ palpable pulse was noted in both the graft and the left above-knee popliteal artery. A 1+ weak pulse was noted at the left posterior tibial artery. The patient recovered excellently, beginning to walk in the unit by the next morning, and was discharged later that evening. Doppler on follow-up two weeks later demonstrated continued patency of the bypass graft and improvement in symptoms. His consent was obtained for publication of this report.

## Discussion

The failure of clinicians to identify many patients with PAD is likely due to a low index of suspicion attributable to the young age of many PAD patients [2]—most PAD patients not having experienced a serious cardiovascular event [3], and only 10% of PAD patients having experienced classic intermittent claudication (IC) [4]. Even amongst the minority of PAD patients experiencing IC, only 10%-50% consult a physician about their symptoms [3]. This is even more common in young adults with PAD, who lead a more active lifestyle and are more likely to attribute their leg pain to benign muscle cramps or spasms rather than a serious health concern, as in our patient. Although the yield of routinely testing ABI in younger, asymptomatic individuals without risk factors is low and not recommended [5], the identification of patients with PAD might be significantly improved by selectively testing ABI in patients of any age without a palpable pedal pulse, namely the posterior tibial which has a 71% sensitivity and 91% specificity for PAD [6], particularly when presenting with any type of leg pain or limited mobility.

There was a four-year delay in our patient’s PAD diagnosis despite presenting with classic IC. Although our case demonstrates that IC in young adults can be caused by atherosclerotic disease, even in the absence of risk factors, other more common arterial and nonarterial causes of IC in young adults should be excluded as management often differs significantly [2]. Tables 1 and 2 list the extensive differential diagnosis highlighting characteristic clinical, epidemiological, and imaging features.

**Table 1:** Differential diagnosis of arterial causes of intermittent claudication.

Arterial pathology	Description	Angiographic findings	Other features
Mural			
Cystic adventitial disease [7]	Cysts in adventitia from mucin-secreting mesenchymal cells; 89% in popliteal artery	Luminal stenosis and cystic changes ± knee joint involvement	Usually unilateral
Fibromuscular dysplasia [8]	Medial fibroplasia in >70%; 5% in external iliac artery	Multifocal stenosis, "string of beads" appearance	Usually unilateral
Vasculitis			
Takayasu arteritis [9]	Large artery intimal proliferation, fibrosis, granulomatosis; 15% in common iliac artery	Mural thickening with long-tapering stenosis and contrast in walls	Usually bilateral Asians
Thrombo-angiitis obliterans [10-11]	Medium-small artery intimal proliferation, fibrosis, granulomatosis; 39% anterior tibial and 38% posterior tibial artery	Multifocal, segmental stenosis with “corkscrew” or “tree root” shaped collaterals	Usually unilateral with ulceration of digits smokers
Compression			
Iliac artery endofibrosis [12]	Intimal proliferation of external iliac ± common femoral artery from hip hyperflexion	Kinking of common iliac with narrowing of proximal external iliac	Usually unilateral cyclists
Popliteal artery entrapment [13]	Compression of popliteal artery in popliteal fossa by gastrocnemius	Soft tissue abnormality and occlusion with plantar/dorsiflexion	Usually bilateral
Osteo-chondroma [14]	About 22.5% from distal femur can compress superficial femoral artery at adductor hiatus	Bone mass causing extrinsic occlusion of superficial femoral artery	Usually unilateral
Thrombophilia			
Inherited or acquired [15]	Hypercoagulable state predisposing to arterial thrombosis	Abnormal tissue enhancement without significant atrophy	Usually unilateral acute course

**Table 2:** Differential diagnosis of nonarterial causes of intermittent claudication.

Nonarterial pathology	Description	Diagnostic test	Other features
Venous claudication [16]	Venous reflux and hypertension causing iliofemoral deep vein thrombosis; claudication in 44%	Compression ultrasound	Usually unilateral with tight bursting sensation relieved by leg-raise
Chronic compartment syndrome [17]	Chronic muscle hypertrophy and hypervolemia causes venous obstruction and nerve irritation; >95% in lower leg	Intra-compartment tissue pressures before and after exercise	Usually bilateral in anterior and lateral compartments runners
Lumbar spinal stenosis [18]	Lumbar extension with walking compresses cauda equina and occludes subarachnoid space causing venous stasis	Magnetic resonance imaging	Bilateral in buttocks and posterior thigh and relieved by lumbar flexion

After confirming a diagnosis of PAD with ABI ± imaging, current guidelines suggest that all patients receive a medical therapy regimen of aspirin or clopidogrel, statin, and angiotensin-converting enzyme inhibitor or angiotensin II receptor blockers. These medications significantly reduce the risk of myocardial infarction, stroke, and vascular death in both symptomatic and asymptomatic PAD patients. In symptomatic patients, statins have the additional benefit of increasing pain-free walking time and distance. Cilostazol and supervised exercise programs should also be added to increase pain-free walking time and distance, although they do not reduce the risk of vascular events [5]. For patients experiencing severe lifestyle-limiting claudication with inadequate response to conservative management, guidelines suggest endovascular or surgical revascularization [5]. After a detailed discussion of risks and benefits, plans for early revascularization were made due to patient preference. Due to his young age and previously high activity level, the patient was primarily concerned with achieving maximal improvement in functional status as quickly as possible. Although both supervised exercise programs and revascularization have comparable short- and long-term benefits and are superior to medical therapy alone, the combination of supervised exercise and revascularization improves walking distance and quality of life more significantly than either therapy alone [19-20]. This plan also carried minimal procedural risk given our patient’s lack of comorbidities and would provide initial functional improvement with revascularization that might improve adherence to a rigorous supervised exercise program.

## Conclusions

Peripheral artery disease is commonly thought to be a disease of the middle age and elderly but can occur in young adults even in the absence of risk factors. Diagnosis requires a high index of suspicion and careful exclusion of other causes of claudication. In severe cases, early revascularization provides a safe and effective treatment option.
